# Supplementary material for: Thiophene-Linked 1,2,4-Triazoles: Synthesis, Structural Insights and Antimicrobial and Chemotherapeutic Profiles
Source: Pharmaceuticals (Basel). 2024 Aug 25;17(9):1123. doi: 10.3390/ph17091123 (PMC11435084; doi:10.3390/ph17091123)
Supplement: Supplementary file 1 [file pharmaceuticals-17-01123-s001.zip › pharmaceuticals-3150023-supplementary.pdf]

Supporting Information

Thiophene-Linked 1,2,4-Triazoles: Synthesis, Structural Insights and Antimicrobial and Chemotherapeutic Profiles

Nada A. El-Emam, Mahmoud B. El-Ashmawy, Ahmed A. B. Mohamed, El-Sayed E. Habib, Subbiah Thamotharan, Mohammed S. M. Abdelbaky, Santiago Garcia-Granda and Mohamed A. A. Moustafa 1

Contents

1. Crystal data and structure refinement parameters for compounds **5a** and **5b** (Table S1).
2. Crystal data and structure refinement parameters for compounds **6a**, **6d** and **10a** (Table S2).
3. Molecular formulae, molecular weights and elemental analyses data of compounds **5a-e**, **6a-e**, **7a-d**, **8**, **9**, **10a** and **10b** (Table S3).
4. Determination of in vitro antimicrobial activity for compounds **5a-e**, **6a-e**, **7a-d**, **8**, **9**, **10a** and **10b** (agar disc-diffusion method).
5. Determination of minimal inhibitory concentrations (MIC) and the minimal bactericidal concentrations (MBC) for compounds **5b-e**, **6a-e**, **7a-d**, **8**, **9** and **10a** (micro-dilution susceptibility method).
6. Determination of in vitro anti-proliferative activity for compounds **5a-e**, **6a-e**, **7a-d**, **8**, **9**, **10a** and **10b** (MTT assay).
7. ^{1}H NMR and ^{13}C NMR spectra.

Table S1. Crystal data and structure refinement parameters for compounds **5a** and **5b**.

	**Compound 5a**	**Compound 5b**
CCDC number	2343567	2343568
Empirical formula	$C_{12}H_8N_3FS_2$	$C_{12}H_7BrFN_3S_2$
Formula weight	277.33	356.24
Crystal system	Triclinic	Triclinic
Space group	*P-1*	*P-1*
Temperature (K)	293 (2)	160 (1)
a, *b*, c (Å)	8.3359 (4), 8.4996 (4), 9.6292 (5)	8.1835 (3), 8.2711 (2), 11.0462 (5)
α, β, γ (°)	89.990 (4), 84.868 (4), 68.268 (4)	104.951 (3), 93.926 (4), 109.018 (3)
Volume (Å^3)	630.82 (6)	673.35 (5)
Z	2	2
ϱcalc (g/cm^3)	1.460	1.757
μ (mm^{-1})	3.818	7.081
Crystal size (mm^3)	0.19 × 0.09 × 0.09	0.24 × 0.13 × 0.04
Diffractometer	Excalibur, Ruby, Gemini	Rigaku OD SuperNova/Atlas area-detector
Radiation type	Cu $K\alpha$ (λ = 1.54184 Å)	Cu $K\alpha$ (λ = 1.54184 Å)
2Θ range for data collection (°)	3.8-74.6°	3.1-71.6°
Index ranges	-10 ≤ h ≤ 10, -10 ≤ k ≤ 10, -11 ≤ l ≤ 11	-10 ≤ h ≤ 10, -10 ≤ k ≤ 10, -13 ≤ l ≤ 13
Reflections collected	9673	12412
Independent reflections	2551	12412
Data/restraints/parameters	2551/0/167	12412/0/178
Goodness-of-fit on F^2	1.043	1.119
Final *R* indexes [I>=2σ (I)]	R_1 = 0.0385, wR_2 = 0.1036	R_1 = 0.0338, wR_2 = 0.1031
Final *R* indexes [all data]	R_1 = 0.0477, wR_2 = 0.1112	R_1 = 0.0357, wR_2 = 0.1071
$\Delta\varrho_{max}/\Delta\varrho_{min}$ (e Å^{-3})	0.23/-0.34	0.40/-0.50

Table S2. Crystal data and structure refinement parameters for compounds **6a**, **6d** and **10a**.

	**Compound 6a**	**Compound 6d**	**Compound 10a**
CCDC number	2343570	2343571	2343572
Empirical formula	$C_{18}H_{19}N_4FS_2$	$C_{17}H_{16}N_4OFS_2Br$	$C_{20}H_{17}N_4FS_2$
Formula weight	374.49	455.37	396.49
Crystal system	Monoclinic	Monoclinic	Monoclinic
Space group	$P2_1/c$	$P2_1/c$	$P2_1/c$
Temperature (K)	119 (2)	293 (2)	108 (2)
a, *b*, c (Å)	5.5622 (3), 18.3760 (10), 35.6899 (18)	16.7781 (3), 12.9005 (2), 8.8975 (2)	10.8829 (1), 5.3713 (1), 32.5870 (4)
α, *β*, *γ* (°)	90, 100.874 (6), 90	90, 92.977 (2), 90	90, 94.703 (1), 90
Volume (Å^3)	3582.4 (3)	1923.23 (6)	1898.47 (5)
Z	8	4	4
ϱcalc (g/cm^3)	1.389	1.573	1.387
μ (mm^{-1})	2.850	5.152	2.726
Crystal size (mm^3)	0.25 × 0.06 × 0.05	0.18 × 0.11 × 0.05	0.20 × 0.16 × 0.13
Diffractometer	Excalibur, Ruby, Gemini	Excalibur, Ruby, Gemini	Excalibur, Ruby, Gemini
Radiation type	Cu *Kα* (λ = 1.54184 Å)	Cu *Kα* (λ = 1.54184 Å)	Cu *Kα* (λ = 1.54184 Å)
2Θ range for data collection (°)	5.042-150.266	5.274-149.768	5.442-149.51
Index ranges	-5 ≤ h ≤ 6, -22 ≤ k ≤ 22, -44 ≤ l ≤ 44	-20 ≤ h ≤ 20, -16 ≤ k ≤ 16, -11 ≤ l ≤ 9	-13 ≤ h ≤ 13, -5 ≤ k ≤ 6, -40 ≤ l ≤ 40
Reflections collected	34694	14835	18812
Independent reflections	7219	3856	3843
Data/restraints/parameters	7219/320/526	3856/160/273	3843/160/282
Goodness-of-fit on F^2	1.017	1.011	1.043
Final *R* indexes [I>=2σ (I)]	R_1 = 0.0578, wR_2 = 0.0947	R_1 = 0.0484, wR_2 = 0.0967	R_1 = 0.0335, wR_2 = 0.0818
Final *R* indexes [all data]	R_1 = 0.1222, wR_2 = 0.1178	R_1 = 0.1021, wR_2 = 0.1231	R_1 = 0.0409, wR_2 = 0.0863
$\Delta\varrho_{max}/\Delta\varrho_{min}$ (e Å^{-3})	0.29/-0.29	0.38/-0.41	0.24/-0.19

Table S3. Molecular formulae, molecular weights and elemental analyses data of compounds **5a-e**, **6a-e**, **7a-d**, **8**, **9**, **10a** and **10b**.

Comp. No.	Mol. Formula (Mol. Wt.)	Analysis: % Calcd. (Found)			
		C	H	N	S
**5a**	$C_{12}H_8FN_3S_2$ (277.34)	51.97 (51.76)	2.91 (2.92)	15.15 (15.10)	23.12 (23.02)
**5b**	$C_{12}H_7BrFN_3S_2$ (356.23)	40.46 (40.38)	1.98 (2.01)	11.80 (11.78)	18.0 (17.98)
**5c**	$C_{12}H_7BrClN_3S_2$ (372.68)	38.67 (38.70)	1.89 (2.01)	11.28 (11.22)	17.20 (17.0)
**5d**	$C_{12}H_7BrClN_3S_2$ (372.68)	38.67 (38.48)	1.89 (2.02)	11.28 (11.25)	17.20 (17.12)
**5e**	$C_{12}H_7Br_2N_3S_2$ (417.14)	34.55 (34.32)	1.69 (1.70)	10.07 (9.88)	15.37 (15.28)
**6a**	$C_{18}H_{19}FN_4S_2$ (374.50)	57.73 (57.48)	5.11 (5.18)	14.96 (14.90)	17.12 (17.13)
**6b**	$C_{17}H_{17}FN_4OS_2$ (376.47)	54.24 (54.31)	4.55 (4.55)	14.88 (14.86)	17.03 (16.95)
**6c**	$C_{17}H_{17}FN_4S_3$ (392.53)	52.02 (51.89)	4.37 (4.43)	14.27 (14.25)	24.50 (24.40)
**6d**	$C_{17}H_{16}BrFN_4OS_2$ (455.36)	44.84 (44.68)	3.54 (3.56)	12.30 (12.28)	14.08 (14.10)
**6e**	$C_{17}H_{16}BrFN_4S_3$ (471.43)	43.31 (43.30)	3.42 (3.50)	11.88 (11.68)	20.40 (20.28)
**7a**	$C_{18}H_{20}FN_5S_2$ (389.51)	55.50 (55.32)	5.18 (5.22)	17.98 (18.0)	16.46 (16.40)
**7b**	$C_{23}H_{22}FN_5S_2$ (451.58)	61.17 (61.17)	4.91 (4.92)	15.51 (15.45)	14.20 (14.08)
**7c**	$C_{23}H_{21}BrFN_5S_2$ (530.48)	52.08 (52.92)	3.99 (4.08)	13.20 (13.06)	12.09 (12.02)
**7d**	$C_{24}H_{23}BrFN_5OS_2$ (560.50)	51.43 (51.39)	4.14 (4.22)	12.49 (12.28)	11.44 (11.34)
**8**	$C_{24}H_{23}FN_4S_2$ (450.59)	63.97 (63.85)	5.15 (5.22)	12.43 (12.41)	14.23 (14.23)
**9**	$C_{22}H_{19}FN_4S_2$ (422.54)	62.54 (62.30)	4.53 (4.58)	13.26 (13.28)	15.17 (15.14)
**10a**	$C_{20}H_{17}FN_4S_2$ (396.50)	60.58 (60.43)	4.32 (4.37)	14.13 (14.0)	16.17 (16.05)
**10b**	$C_{26}H_{21}FN_4S_2$ (472.60)	66.08 (59.97)	4.48 (4.52)	11.86 (11.53)	13.57 (13.55)

Determination of in vitro antimicrobial activity of compounds 5a-e, **6a-e**, **7a-d**, **8**, **9**, **10a** and **10b (agar disc-diffusion method)**

Sterile filter paper discs (8 mm diameter) were moistened with compounds **5a-e**, **6a-e**, **7a-d**, **8**, **9**, **10a** and **10b** solution in dimethyl sulfoxide of specific concentration (200 μg/disc), the broad-spectrum antibacterial drugs Ampicillin trihydrate, Ciprofloxacin and the antifungal drug Fluconazole (100 μg/disc) were carefully placed on the agar culture plates that had been previously inoculated separately with the microorganisms. The plates were incubated at 37 °C, and the diameter of the growth inhibition zones were measured after 24 hours in case of bacteria and 48 hours in case of fungi.

Determination of minimal inhibitory concentrations (MIC) and the minimal bactericidal concentrations (MBC) for compounds 5a, 5b, 5e, 6b-e, 7a-d, 8, 9 and **10a (micro-dilution susceptibility method)**

Compounds **5a**, **5b**, **5e**, **6b-e**, **7a-d**, **8**, **9** and **10a**, Ampicillin trihydrate and Ciprofloxacin were dissolved in dimethyl sulfoxide at concentration of 128 μg/mL. Two-fold dilutions of the solution were prepared (128, 64, 32, …, 0.5 μg/mL). The microorganism suspensions at 10^6 CFU/mL (colony forming unit/ml) concentrations were inoculated to the corresponding wells and the plates were incubated at 36 °C for 24 hours. The MIC values were determined as the lowest concentration that completely inhibited visible growth of the microorganism as detected by unaided eye. The MBC values were determined by the lowest concentration that killed of the microorganism by re-cultured on agar medium to verify the absence of growth.

Determination of in vitro anti-proliferative activity for compounds 5a-e, **6b-e**, **7a-d**, **8**, **9** and **10a** and **10b (MTT assay)**

The tumor cells (3000 cells per well) were cultured and seeded into 96-well plates and the plates were incubated for 24 hours. The cells were then treated with compounds **5a-e**, **6b-e**, **7a-d**, **8**, **9** and **10a** and **10b** and Doxorubicin at different concentrations in dimethyl sulfoxide (0.1 μM to 100 μM) at 37 °C in an atmosphere of 5% CO_2 for 48 hours. Freshly prepared 3-[4,5-dimethylthiazoyl-2-yl]-2,5-diphenyltetrazolium bromide (MTT) was added to each well at a terminal concentration of 5 μg/mL and incubated with cells at 37 °C for 4 hours. The formazan crystals were dissolved in 100 μL of dimethyl sulfoxide in each well, and the absorbency at 492 nm (for absorbance of MTT formazan) and 630 nm (for the reference wavelength) was measured with an enzyme linked immunosorbent assay (ELISA) reader (ChroMate-4300, FL, USA). All compounds were tested three times in each of the cell lines. The IC_{50} values were calculated according to the equation for Boltzmann sigmoidal concentration response curve using the nonlinear regression fitting

models (Graph Pad, Prism Version 5). The results reported are means of three separate experiments. Statistical differences were analyzed according to one-way ANOVA test wherein the differences were considered to be significant at $p < 0.05$.

^{1}H NMR and ^{13}C NMR spectra

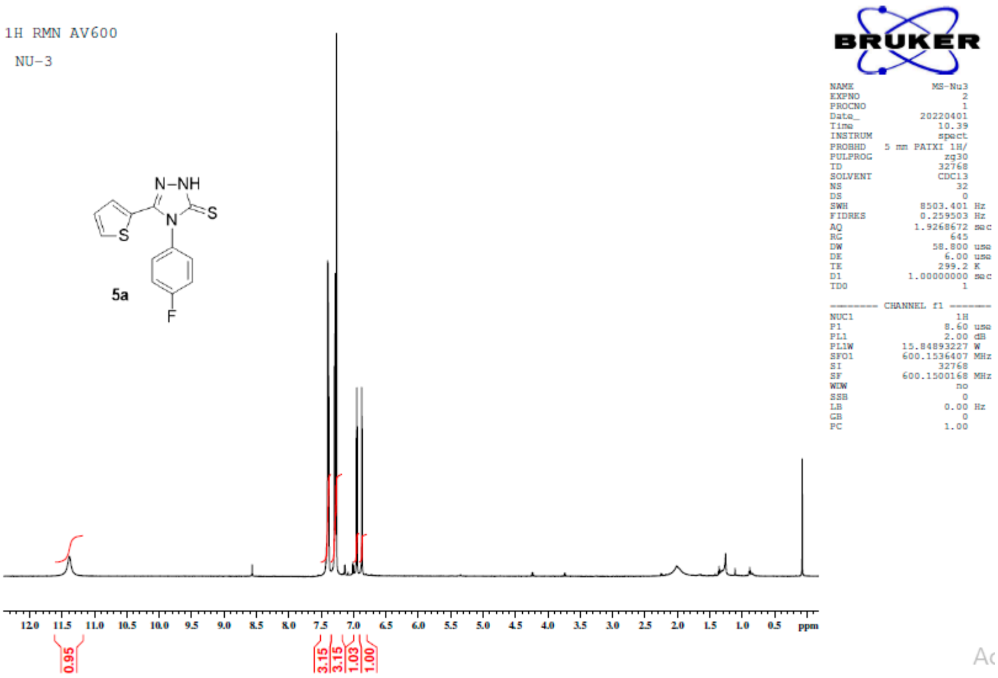

^{1}H NMR (600.15 MHz) of compound **5a**

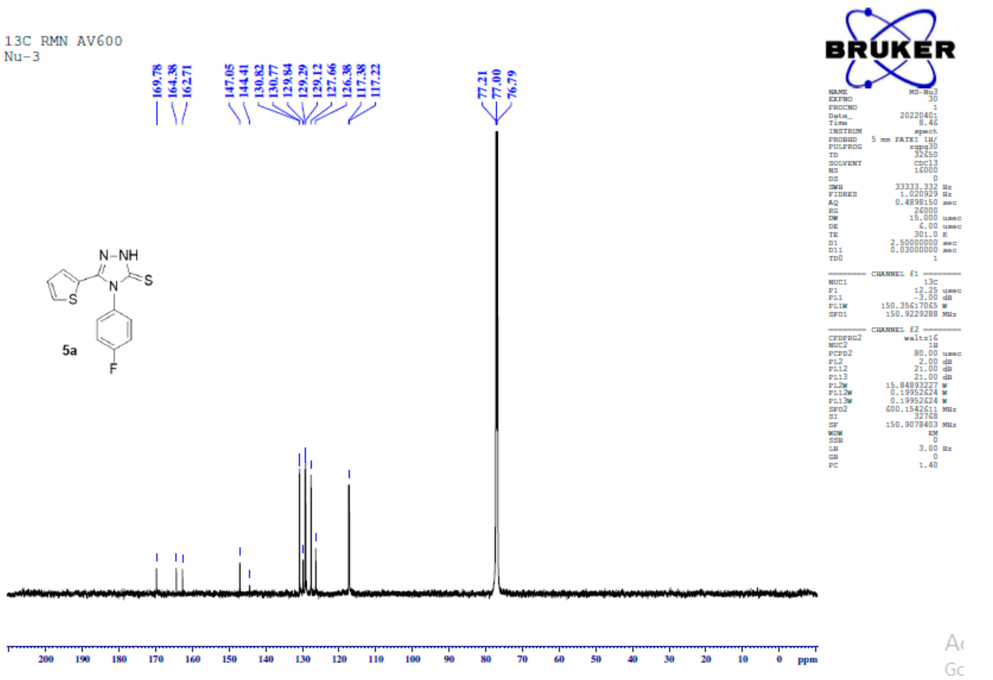

^{13}C NMR (150.91 MHz) of compound **5a**

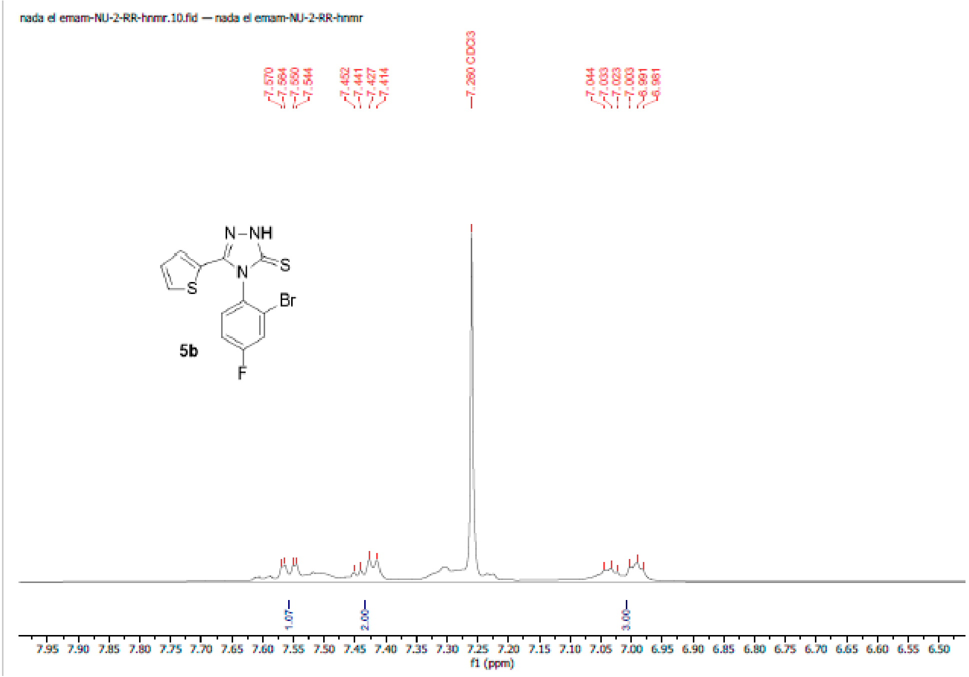

^{1}H NMR (400.20 MHz) of compound **5b**

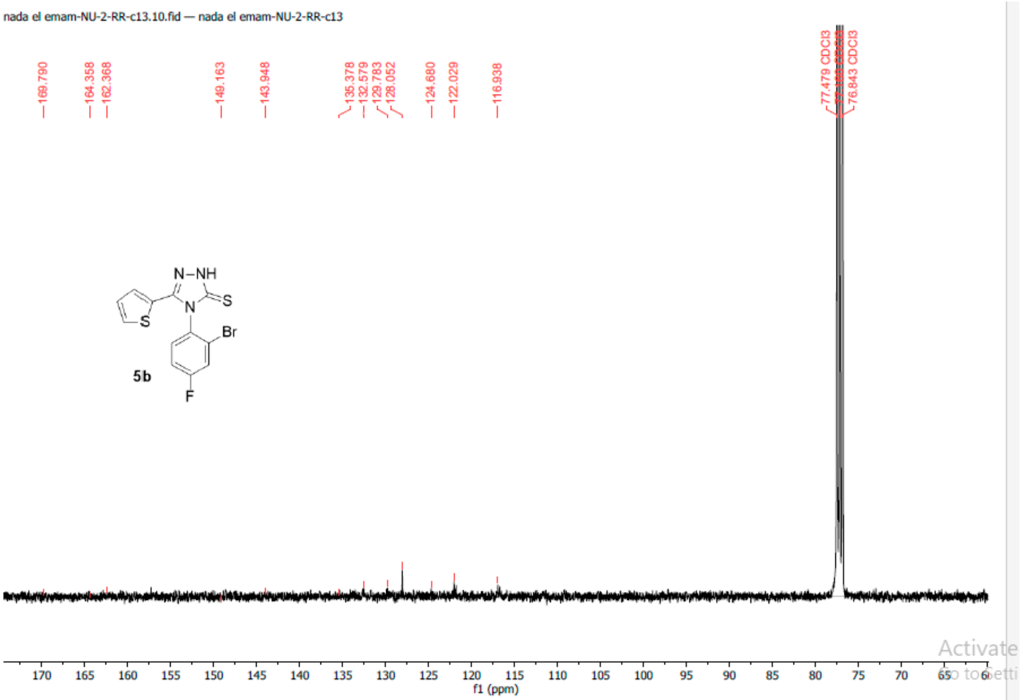

^{13}C NMR (100.64 MHz) of compound **5b**

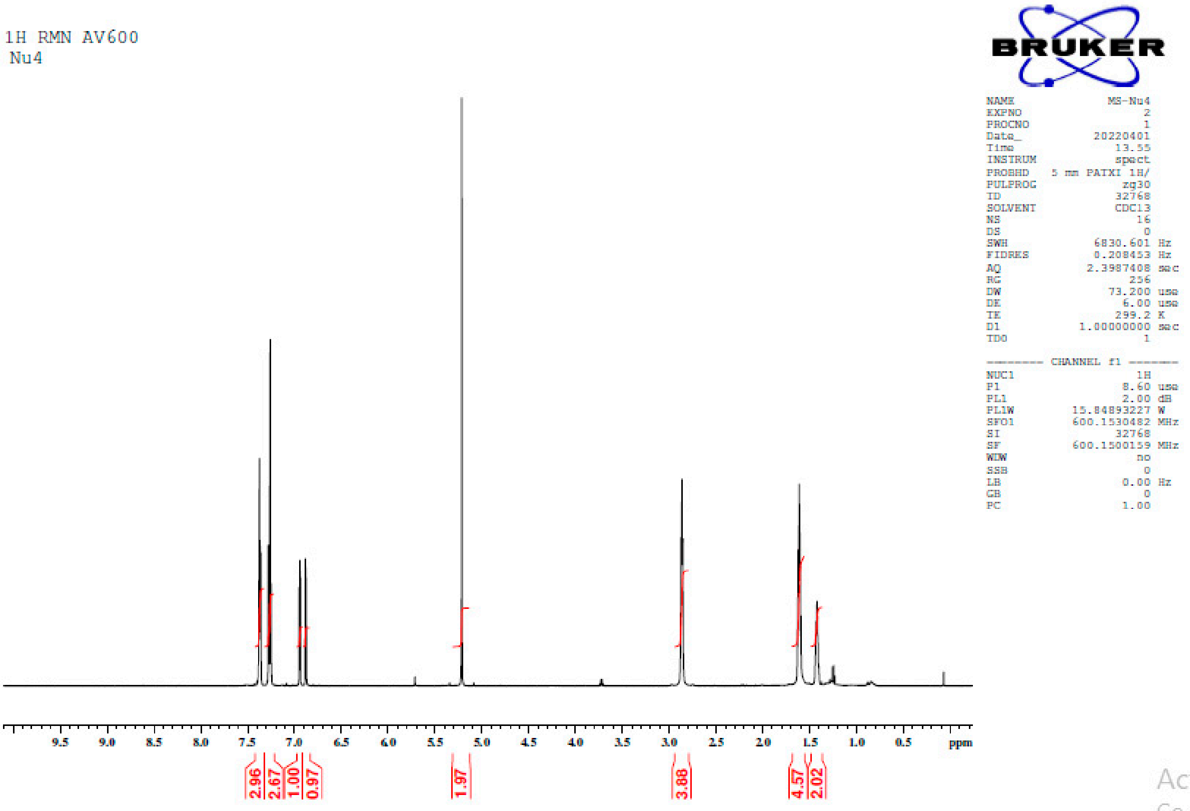

^{1}H NMR (600.15 MHz) of compound **6a**

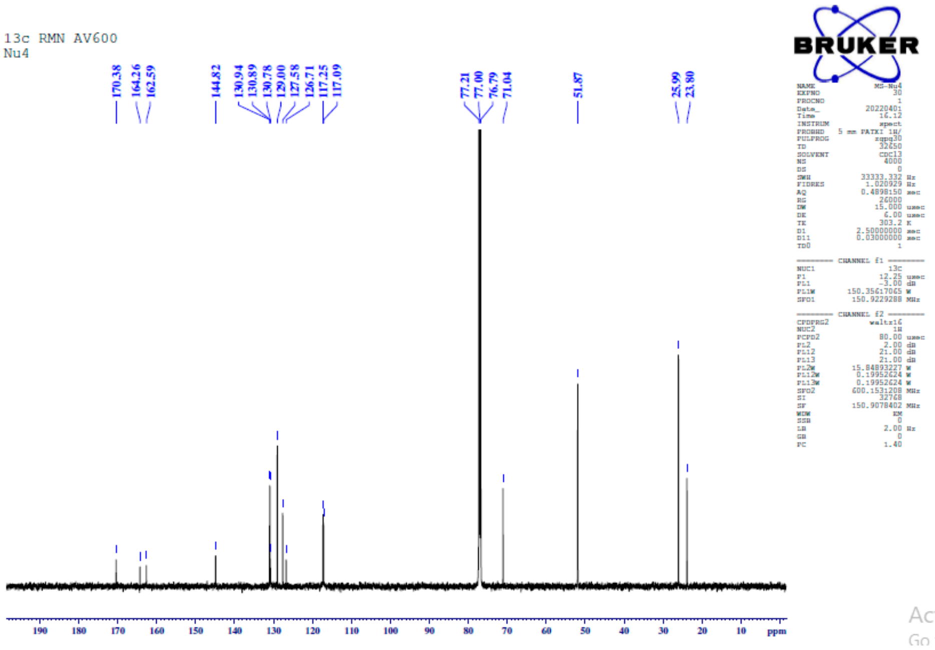

^{13}C NMR (150.36 MHz) of compound **6a**

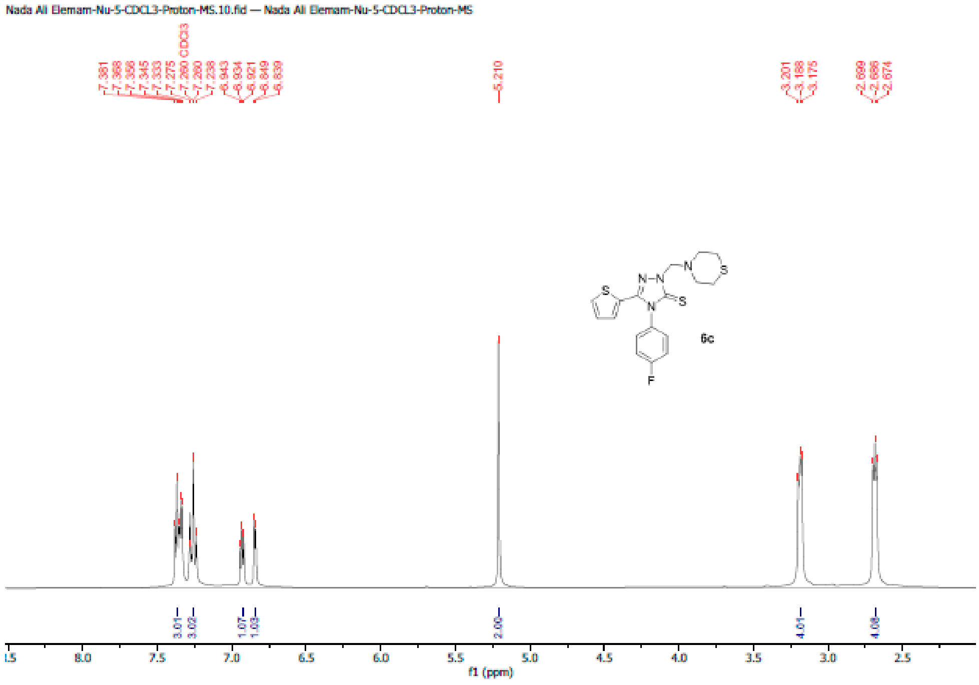

^{1}H NMR (400.20 MHz) of compound **6c**

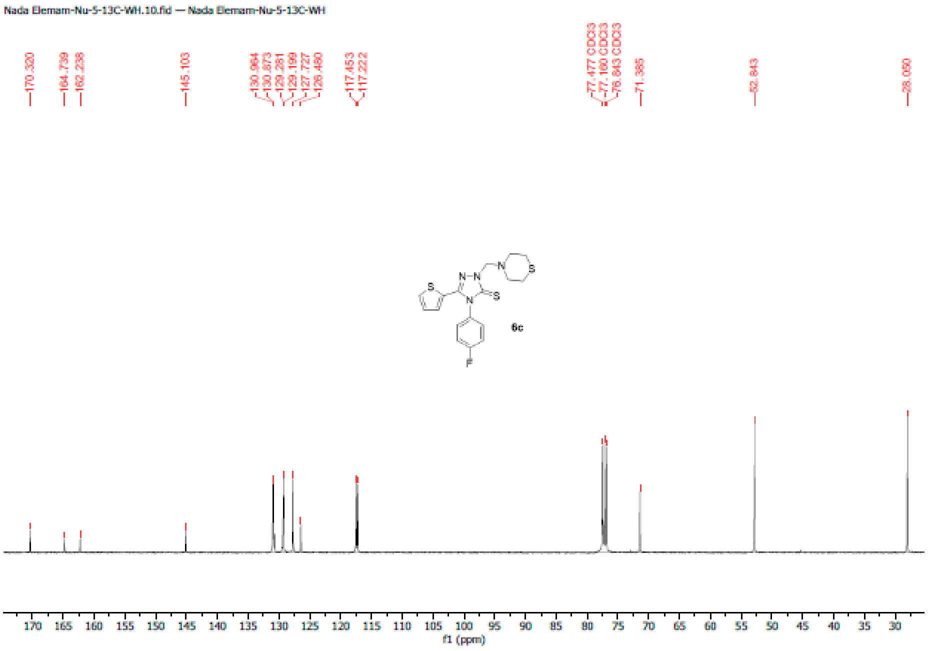

^{13}C NMR (100.64 MHz) of compound **6c**

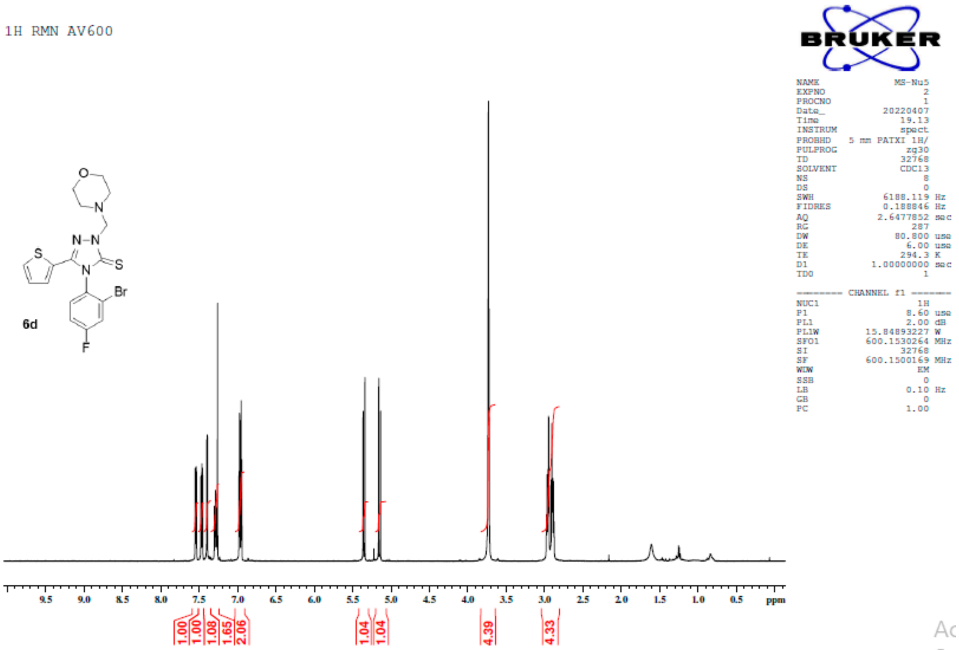

^{1}H NMR (600.15 MHz) of compound **6d**

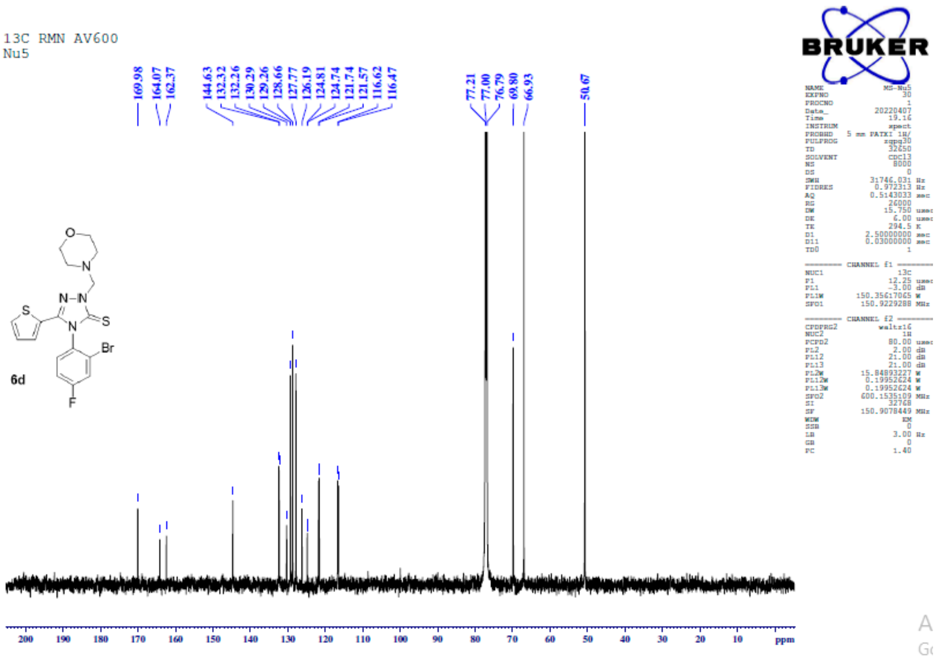

^{13}C NMR (150.36 MHz) of compound **6d**

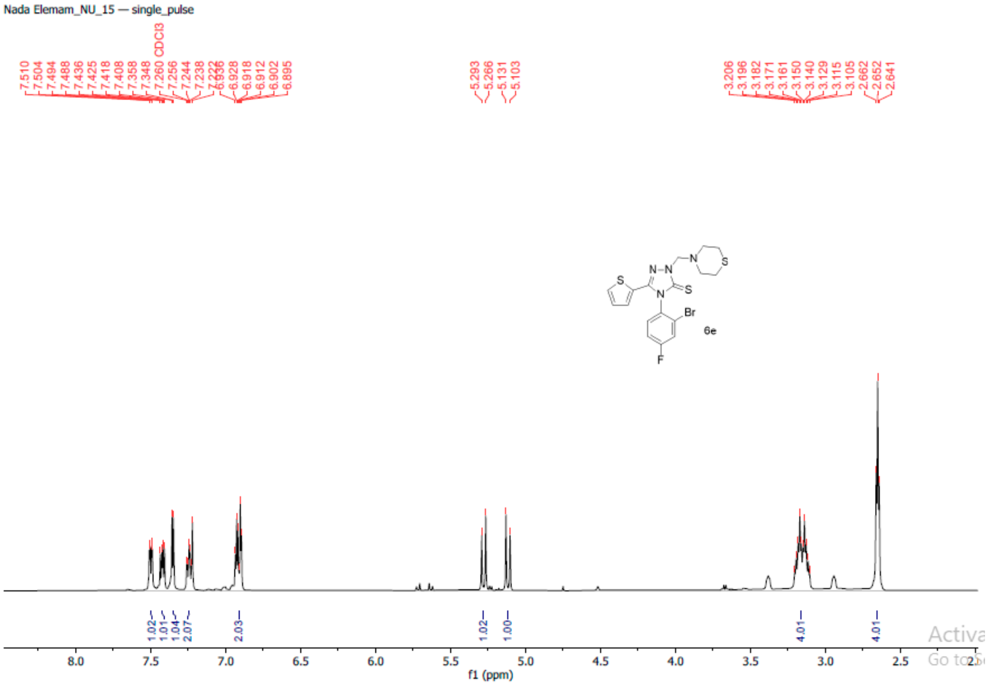

^{1}H NMR (400.20 MHz) of compound **6e**

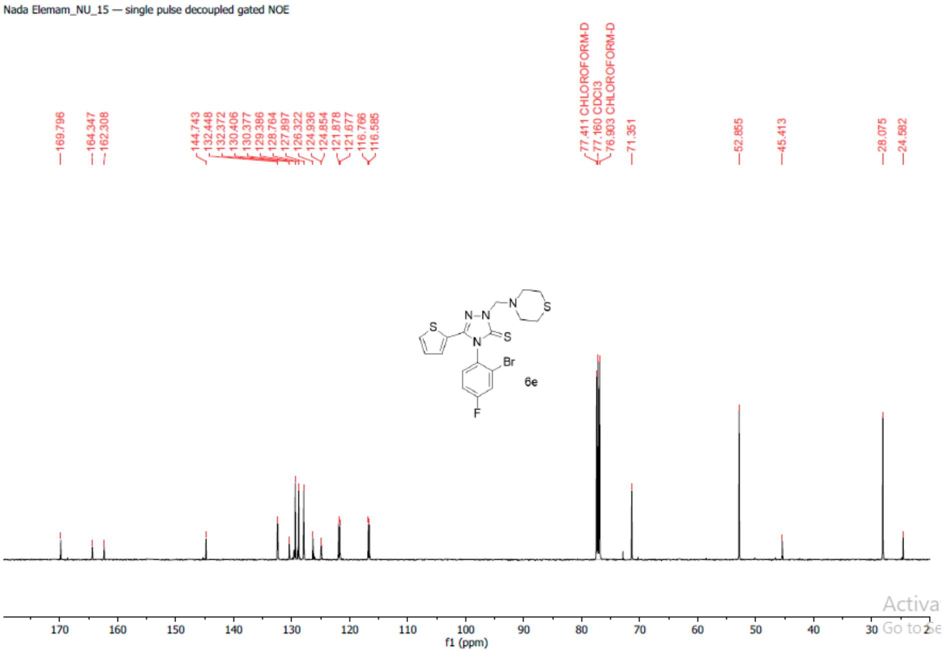

^{13}C NMR (100.64 MHz) of compound **6e**

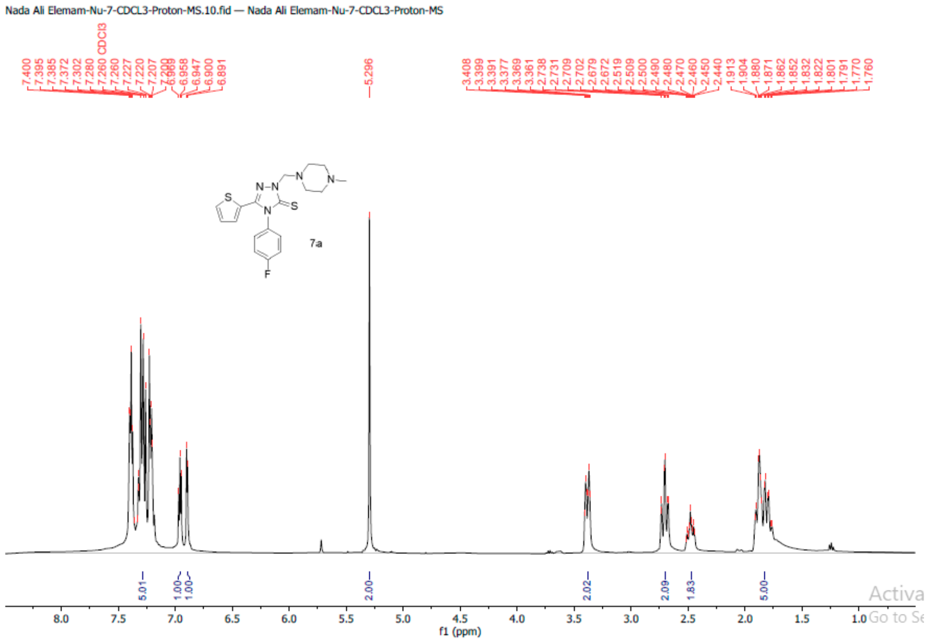

^{1}H NMR (400.20 MHz) of compound **7a**

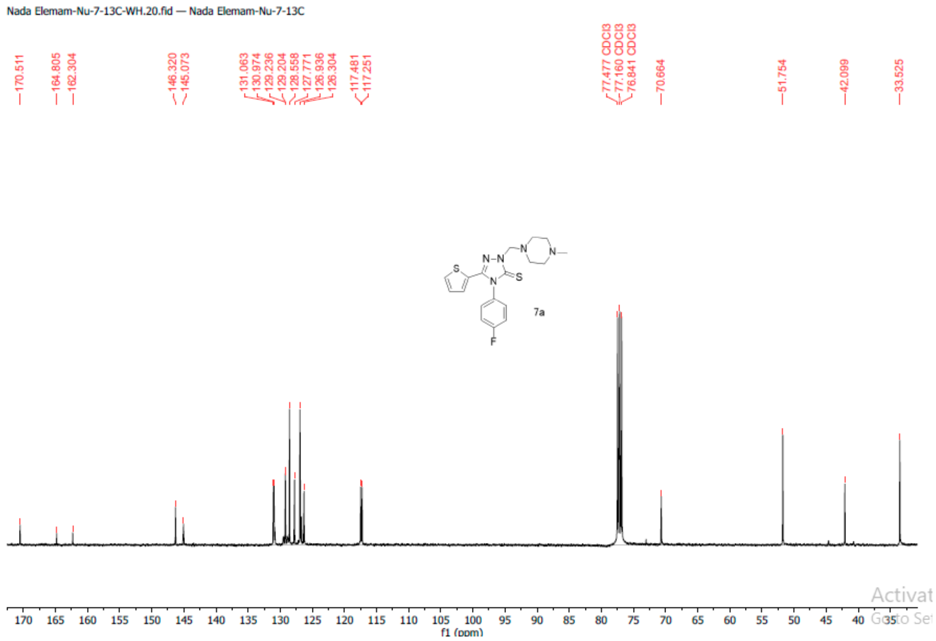

^{13}C NMR (100.64 MHz) of compound **7a**

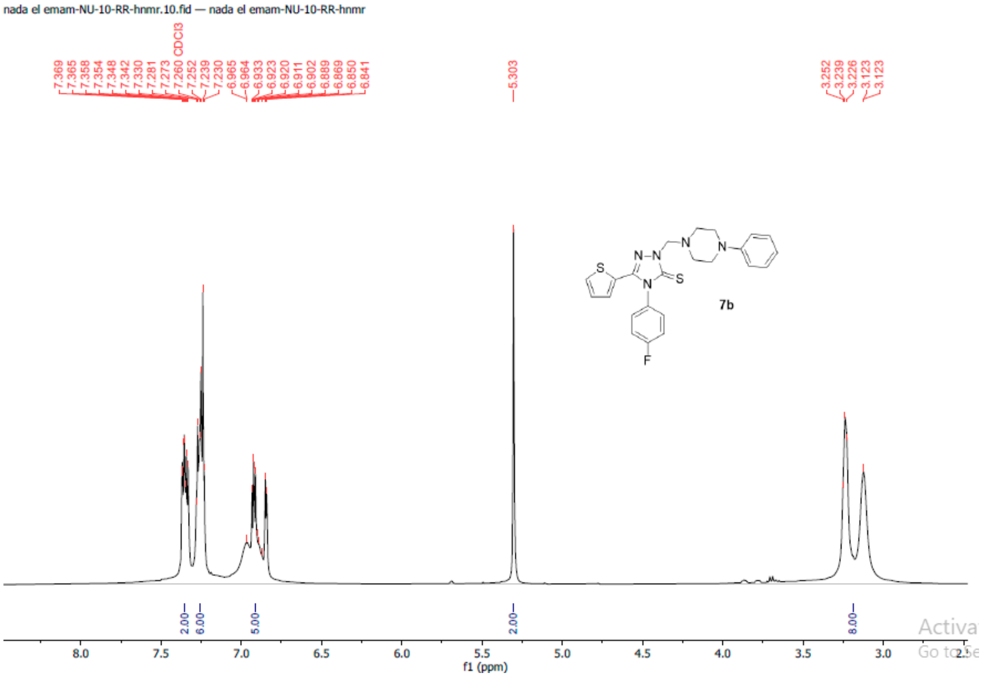

^{1}H NMR (400.20 MHz) of compound **7b**

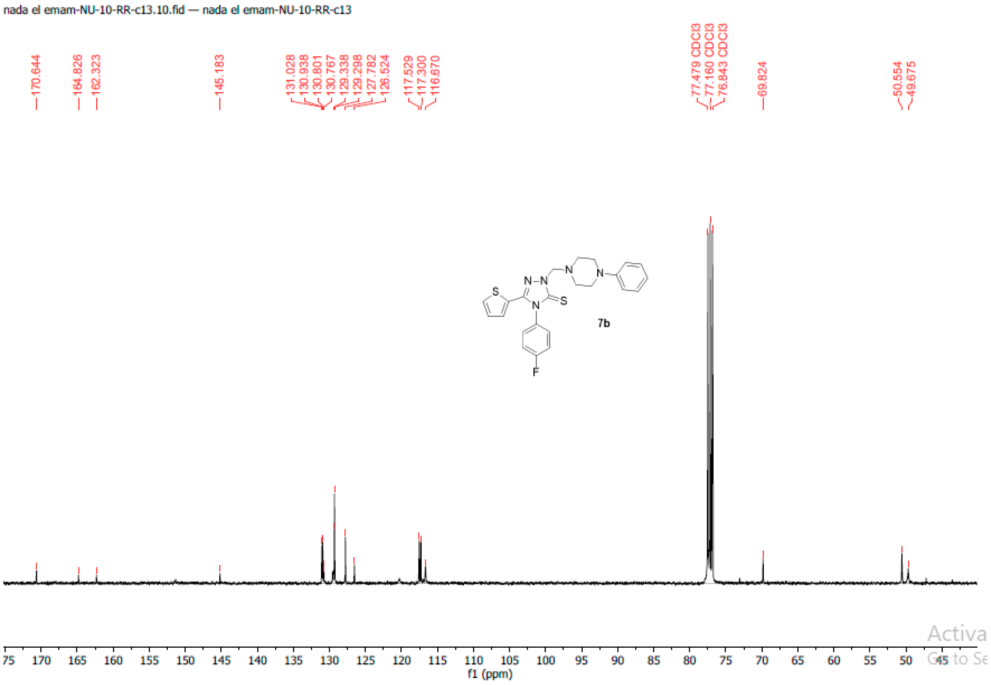

^{13}C NMR (100.64 MHz) of compound **7b**

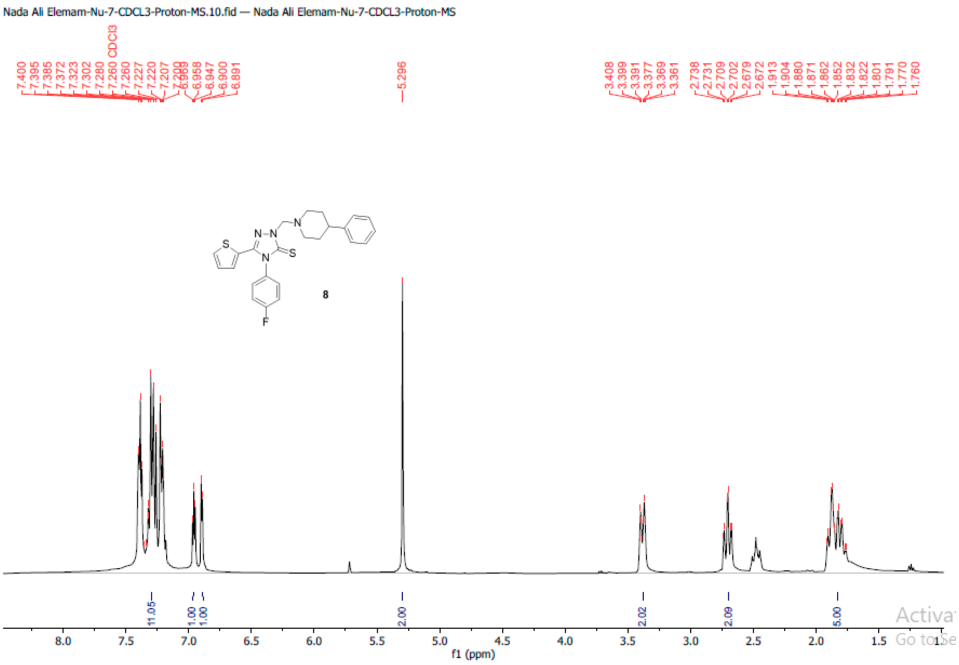

^{1}H NMR (400.20 MHz) of compound **8**

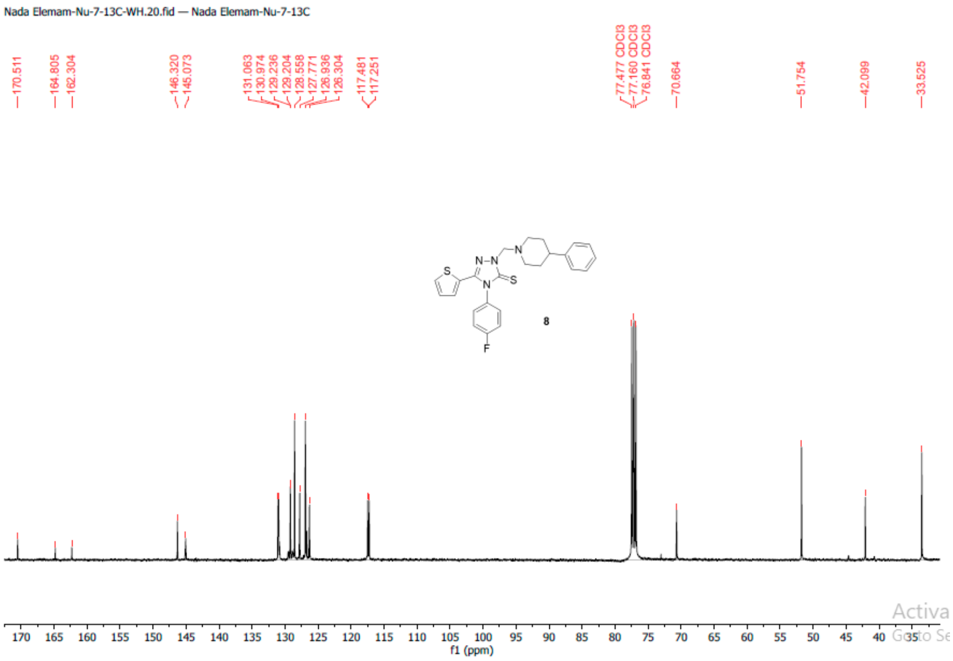

^{13}C NMR (100.64 MHz) of compound **8**

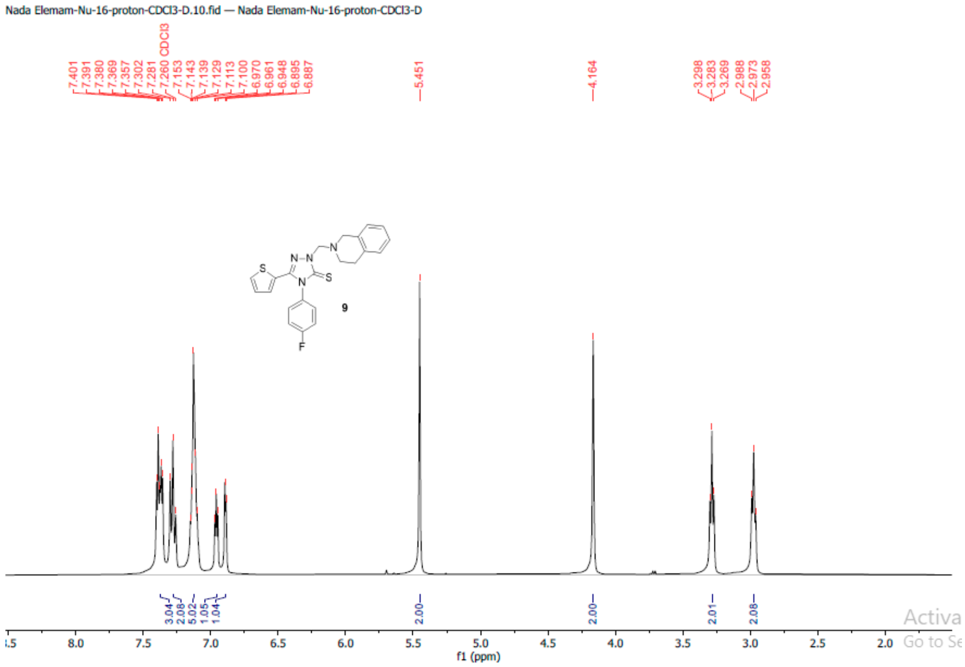

^{1}H NMR (400.20 MHz) of compound **9**

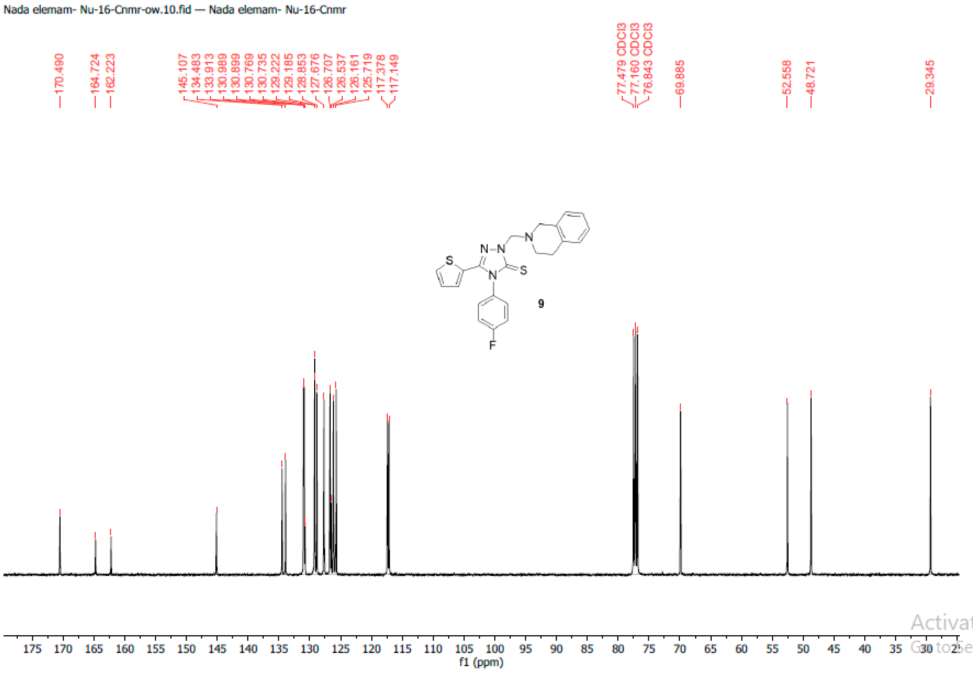

^{13}C NMR (100.64 MHz) of compound **9**

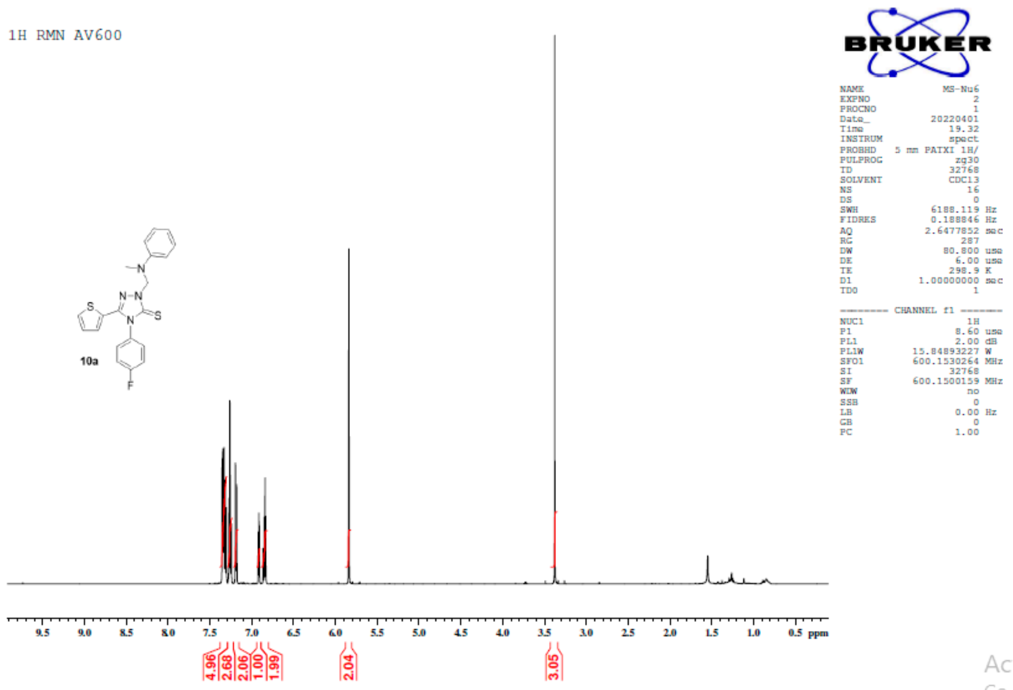

^{1}H NMR (600.15 MHz) of compound **10a**

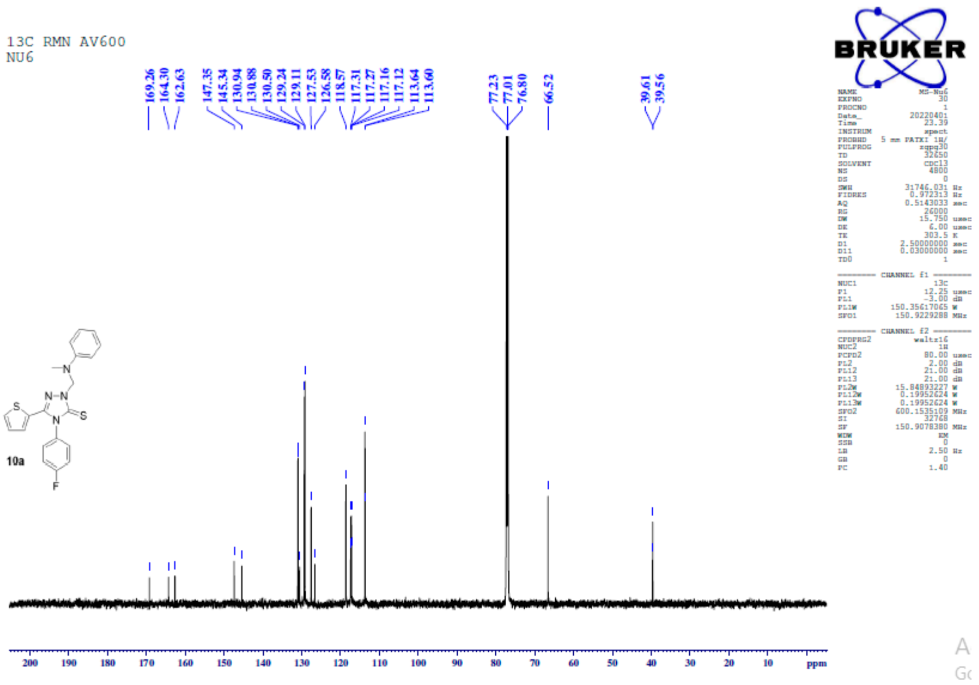

^{13}C NMR (150.36 MHz) of compound **10a**

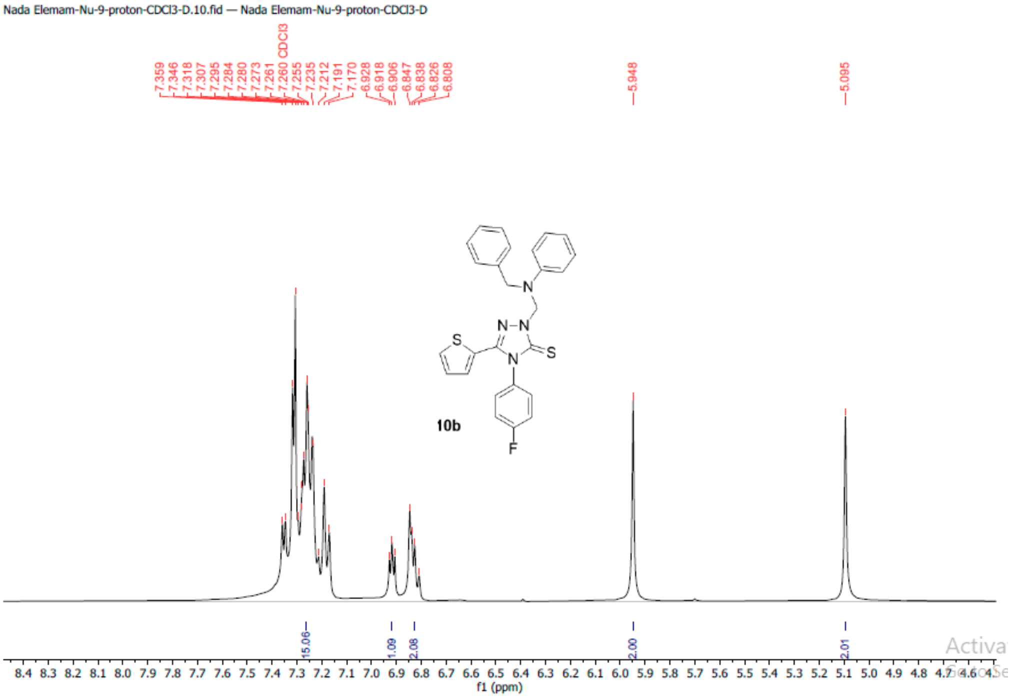

^{1}H NMR (400.20 MHz) of compound **10b**

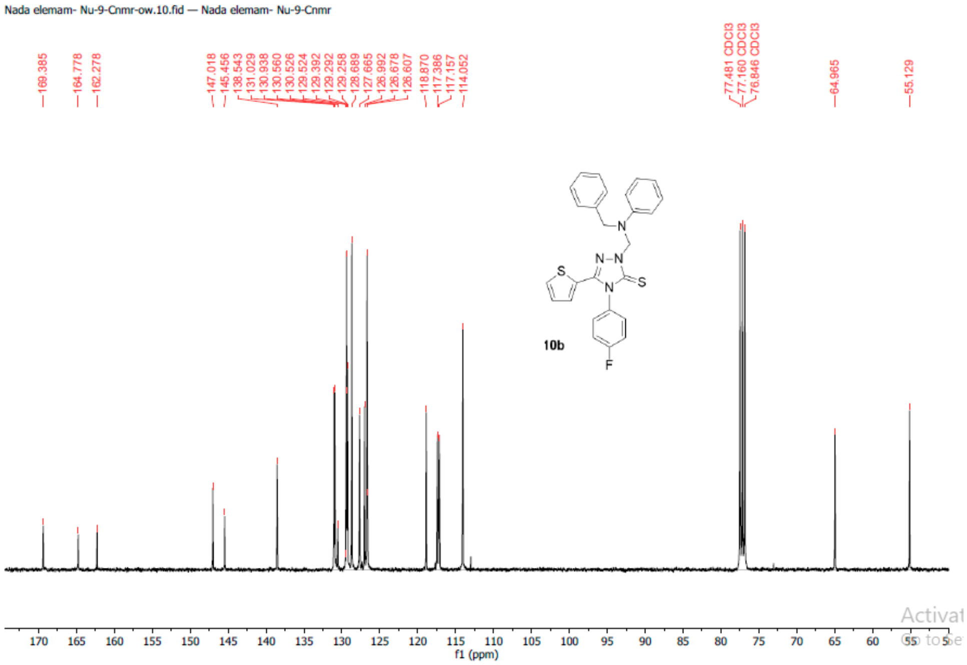

^{13}C NMR (100.64 MHz) of compound **10b**
